# The role of major and minor structural proteins of porcine reproductive and respiratory syndrome virus in induction of protective immunity

**DOI:** 10.3389/fmicb.2025.1563186

**Published:** 2025-03-19

**Authors:** Dan Li, Laixu Zhu, Chenchen Cui, Zhenchun Wu, Pengkai Qing, Qiongqiong Zhou, Peng Gao, Yongning Zhang, Lei Zhou, Xinna Ge, Xin Guo, Jun Han, Hanchun Yang

**Affiliations:** ^1^State Key Laboratory of Veterinary Public Health and Safety, College of Veterinary Medicine, China Agricultural University, Beijing, China; ^2^Key Laboratory of Animal Epidemiology of Ministry of Agriculture and Rural Affairs, College of Veterinary Medicine, China Agricultural University, Beijing, China

**Keywords:** porcine reproductive and respiratory syndrome virus, structural proteins, homologous protection, pathogenicity, vaccine development

## Abstract

**Introduction:**

Porcine reproductive and respiratory syndrome virus (PRRSV), an economically significant threat to the world pork production, is notoriously known for its heterogeneity, and therefore the current vaccines often fail to provide efficient cross-protection against diverse PRRSV strains.

**Methods:**

By making chimeric viruses using HP-PRRSV-2 lineage 8 (JXwn06) and lineage 1 NADC30-like strains (CHsx1401) as model organisms, the recently results have shown that the viral structural protein-coding region is critical for induction of homologous immunity. In this study, the chimeric viruses were further constructed by exchanging the region coding for the minor (GP2/3/4) or major (GP5/M) structural proteins of JXwn06 on the backbone of CHsx1401 to generate two mutants CHsx1401-GP234_JX_ and CHsx1401-GP5M_JX_.

**Results:**

The subsequent animal experiment showed that all three chimeras could confer good protective immunity against the lethal challenge by HP-PRRSV strain JXwn06, and the survived pigs had much lower lung lesions, faster viremia clearance, and lower viral tissue load. However, the exchange of SP region as a whole performed better than either GP2/3/4 or GP5/M region alone, as the pigs in the latter groups showed transient fever following challenge and higher viral load in certain tissues, highlighting a synergistic role. Interestingly, as compared to the group CHsx1401-GP234_JX_, the group CHsx1401-GP5M_JX_ showed excellent viremia clearance, comparable to the SP group.

**Discussion:**

Our results in this report revealed the important role of ORFs2-4 and ORFs5-6 regions in induction of protective immunity and have important implications in understanding viral pathogenesis and further vaccine development.

## Introduction

Porcine reproductive and respiratory syndrome virus (PRRSV) has remained one of the greatest challenges in modern times to the global pig industry (Zhou et al., 2014; Zhang et al., 2022). This virus emerged almost simultaneously for the first time in both North America and Europe in late 1990s, and the associated diseases in the field are typically manifested as reproductive disorder in sows, respiratory distresses in young piglets, and frequent secondary bacterial infections (Dea et al., 2000; Hanada et al., 2005; Sánchez-Carvajal et al., 2022). As of today, many countries have maintained PRRSV epidemic status, leading to enormous economic losses (Kuhn et al., 2016; Han et al., 2017). As a single-stranded, positive-sense RNA virus, PRRSV is a member of the family *Arteriviridae* within the order *Nidovirales* and has a genomic size of about 15 kb that harbors at least 10 open reading frames (ORFs) (Lunney et al., 2010; Zhang et al., 2017; Zhou et al., 2021). Of them, ORF1 encodes viral replicase polyproteins, while ORFs2-7 code for structural proteins (SP) (Kappes and Faaberg, 2015; Zhang et al., 2021). In particular, ORFs2-4 specify the minor structural proteins GP2a, GP3, GP4, and the ion channel protein E (Wissink et al., 2005; Tian et al., 2012; Sun et al., 2013; Luo et al., 2023), whereas ORFs5-6 specify the major structural proteins GP5 and M and a newly discovered protein GP5a with unknown function (Fang and Snijder, 2010; Firth et al., 2011; Johnson et al., 2011). The last ORF7 expresses the viral nucleocapsid protein N (Li et al., 2015; Liu et al., 2018). Among these proteins, GP5 interacts with M to form heterodimers that are necessary for viral attachment, whereas GP2, GP3 and GP4 assemble into heterotrimers that enable virus penetration via engagement with the entry receptor CD163 (Zhou et al., 2009; Lei et al., 2024; Bai et al., 2025; Lim et al., 2025).

The PRRSV is notoriously known for its rapid genetic variation and evolution in the field (Yu et al., 2020). This property has led to the emergence of numerous variants and the quick expansion of viral genetic diversity. For example, in the past 35 years, the PRRSV-2 strains (previous North American type) have evolved into at least 9 lineages via phylogenetic analyses (Han et al., 2006; Brockmeier et al., 2012; Cui et al., 2022). In China, the years 2006–2015 have seen the havoc epidemic of lineage strains, represented by the Chinese highly pathogenic strains (HP-PRRSV), a group of viruses (e.g., JXwn06, HuN4, JXA1, etc.) that had ever devastated the Chinese swine industry (Han et al., 2017). While in recent years, they gradually give away the center stage to the lineage 1 NADC30/34-like strains (e.g., CHsx1401, SD17-38, Anheal-1, etc.), which show a clear genetic divergence greater than 20% at nucleotide level as compared to HP-PRRSV (Bao and Li, 2021; Yuan et al., 2022). Not surprisingly, the current modified live vaccines (MLV) fail to deliver efficient cross-protection against these rising strains (Li et al., 2014). The widespread application of MLV has also led to the selection of frequent recombinants between HP-PRRSV and NADC30/34 strains that potentially enable the virus to escape the preexisting immunity (Sun et al., 2016; Tian et al., 2017; Wang et al., 2018; Cao et al., 2022). To dissect the mechanisms behind the clinical observations, our recent studies have shown that the genetic variation of PRRSV nsp2 replicase protein-coding region is a key factor for modulating viral virulence and persistence, thus providing an explanation on the rise of NADC30 strains in the field, whereas the viral structure protein region (SP) is necessary for inducing efficient homologous immunity via the approach of constructing chimeric viruses (Kong et al., 2023a,b).

This report is a follow-up of our previous work and aims to assess the role of the specific SP regions in induction of homologous immunity. This study started with constructing chimeric mutants by exchanging the region coding for minor glycoproteins (GP2/3/4) or the major glycoproteins (GP5/M) from lineage 8 HP-PRRSV strain JXwn06 in the backbone of lineage 1 NADC30-like strain CHsx1401, followed by animal experiments. Our findings showed that the GP2/3/4 or GP5/M regions work synergistically, while CHsx1401 carrying the whole SP region of JXwn06, but not wild-type CHsx1401, provides the best protection efficacy against the challenge by JXwn06. These findings provide further insight into the structural proteins of PRRSV and their role in providing homologous protection for pigs against HP-PRRSV infection, which has great implications for development of novel PRRSV vaccines. The details are described below.

## Materials and methods

### Cells and viruses

Primary porcine alveolar macrophages (PAMs) derived from 28-day-old specific pathogen free (SPF) piglets were cultured in RPMI 1640 (Gibco, 61870044) containing 10% fetal bovine serum (FBS; Gibco, 16140071) and 1% penicillin (50 U/mL) and streptomycin (50 mg/mL) at 37°C with 5% CO_2_. MARC-145 cells (African green monkey kidney epithelial cells, ATCC, CRL-12231) were maintained in Dulbecco’s modified Eagle’s medium (DMEM; Gibco, 12491015) containing 10% fetal bovine serum (FBS; Gibco, 16140071) and 1% penicillin (50 U/mL) and streptomycin (50 mg/mL) at 37°C with 5% CO_2_. The Chinese highly pathogenic PRRSV strain JXwn06 (GenBank accession number: EF641008.1) and the NADC30-like strain CHsx1401 (GenBank accession number: KP861625.1) used in this study have been described previously (Zhou et al., 2009; Zhou et al., 2015).

### Construction of chimeric PRRSV mutants

The full-length infectious genomic clone plasmids pWSK-CHsx1401 and pWSK-JXwn06, including a cytomegalovirus (CMV) promoter, a hepatitis delta virus ribozyme sequence, and an SV40 polyadenylation signal, was successfully constructed, respectively. To facilitate the exchange of the GP2, GP3, and GP4 or GP5 and M regions, the corresponding genomic region sequences from pWSK-JXwn06, which served as the donor, along with the up-and downstream sequences of this region, were individually amplified using PCR with KOD DNA polymerase (TOYOBO, #KFX-101). The resulting products were purified and then cloned into the backbone plasmid pWSK-CHsx1401 that are double digested with the *Asc*I and *Pac*I restriction enzymes using a homologous recombination strategy with the ClonExpress MultiS one-step cloning kit (Vazyme, C113-02). This process ultimately led to the creation of the infectious clone plasmids pCHsx1401-GP234_JX_ and pCHsx1401-GP5M_JX_. The primers used for the construction of the chimeric clones are listed in Supplementary Table S1. All constructs underwent verification through DNA sequencing and were prepared using the PureYield plasmid miniprep system (Promega, A2492).

### Recovery of chimeric viruses

The infectious cDNA clone plasmids were transfected into MARC-145 cells utilizing the LTX transfection reagent (Invitrogen, 15338100) following to the manufacturer’s instructions. Briefly, MARC-145 cells were cultured and transfected with plasmid at a density of approximately 80% in 6-well plates. The virus-induced cytopathic effect (CPE) was monitored daily, and the whole cell culture was harvested at 7 days post-transfection approximately. The rescued viruses were confirmed by indirect immunofluorescence assay (IFA) using the PRRSV N protein monoclonal antibody SDOW17 (Rural Technologies, United States). Finally, the RNA samples of chimeric viruses were subsequently extracted, reversed transcribed, and subjected to PCR for further verification through sequencing.

### Growth kinetics analysis

PAMs or MARC-145 cells seeded in 12-well plates were infected with the indicated chimeric viruses at an MOI of 0.1. After incubation at 37°C for 1 h, the cells were washed three times with medium to remove unbound viruses and were then supplemented with either RPMI 1640 or DMEM containing 2% FBS, respectively. At the specified time points post-infection, the medium and cells were harvested for viral titration using an endpoint dilution assay, as described previously (Song et al., 2019). Virus titers were expressed as 50% tissue culture infective dose per mL (TCID_50_/mL).

### Animal experiments

A total of twenty-eight 28-day-old SPF piglets were procured from Shiji Pig Breeding & Management facility located in Shouyang County, China. All piglets were tested negative for PRRSV, pseudorabies virus, porcine circovirus type 2, classic swine fever virus, and African swine fever virus using reverse transcription polymerase chain reaction (RT-PCR) and commercial enzyme-linked immunosorbent assay (ELISA) kits for antibodies detection. Animals were housed in separate isolation rooms within the animal facility and were given a three-day acclimatization period prior to the commencement of the experiments. The piglets were randomly divided into 6 groups: CHsx1401 (*n* = 5), CHsx1401-SP_JX_ (*n* = 5), CHsx1401-GP234_JX_ (*n* = 5), CHsx1401-GP5M_JX_ (*n* = 5), DMEM (*n* = 5) and a mock group (*n* = 3). Piglets in the experimental groups received intranasal administration of CHsx1401, CHsx1401-SP_JX_, CHsx1401-GP234_JX_, or CHsx1401-GP5M_JX_ at a dosage of 2 × 10^5^ TCID_50_, while those in the DMEM group were inoculated with DMEM. On the 42 DPI, except for mock group, all immunized piglets were challenged with wild-type JXwn06 strain at a dosage of 2 × 10^6^ TCID_50_.

Animals were monitored for clinical signs and rectal temperatures daily. The detailed scoring system was implemented according to the method described previously (Li et al., 2014). Briefly, the clinical sign scoring system included gross clinical score (GCS), respiratory clinical score (RCS), and nervous signs score (NSS). The piglets’ weights were recorded weekly to calculate the average daily gain (ADG). Blood samples were collected at specified intervals to evaluate viremia and isolate peripheral blood mononuclear cells (PBMCs). On the 21 DPC (days post challenge), animals were euthanized, and the lungs, tonsils, inguinal lymph nodes (ILNs), and submandibular lymph nodes (SLNs) were collected and stained with hematoxylin and eosin (H&E) for histopathological examination. Gross lung lesions were blindly evaluated, with gross pathology scored on a scale of 100 based on the lesion area. The accessory lobes were assigned 5 points each and 27.5 points (15 for dorsal and 12.5 for ventral) for each of the right and left caudal lobes to reach a total of 100 points. According to the severity of the interstitial pneumonia, the lung microscopic lesions were blindly evaluated with a scale ranging from 0 to 4 (0, no microscopic lesions; 1, mild; 2, moderate multifocal interstitial pneumonia; 3, moderate diffusive interstitial pneumonia; 4, severe interstitial pneumonia).

### Isolation of peripheral blood mononuclear cells (PBMCs)

Blood samples were collected using heparin anticoagulant tubes at specified time intervals post-infection, and PBMCs were isolated using Ficoll–Hypaque density gradient centrifugation according to the manufacturer’s instructions (TBD science, LTS1110). The cell deposit was resuspended in Cryostar freezing medium (CELLSAVING_TM_, C40100) and stored in liquid nitrogen for future applications.

### Serum neutralization assay

Heat inactivated serum samples were diluted at two-fold series with DMEM and subsequently incubated with an equal volume (50 μL) of the corresponding virus at a concentration of 100 TCID_50_ at 37°C for 1 h. Afterwards, the mixture was incubated with MARC-145 cells at 37°C for another 1 h. The cells were then washed three times with PBS, after which fresh DMEM media supplemented with 2% FBS was added. After a 24 h incubation period, PRRSV-positive cells were detected using IFA with PRRSV N protein antibody. The titer of neutralizing antibodies (NA) was calculated using the Reed-Muench method.

### Quantitative PCR (qPCR)

To quantify PRRSV chimeric viruses RNA loads in tissues, total RNAs were extracted with TRIzol (Thermo Fisher, 15596026) according to the manufacturer’s instructions. The cDNAs were synthesized by reverse transcription using the FastKing reverse transcription kit (Tiangen, KR116) with the indicated primers (Supplementary Table S2). To quantify the viral tissue load, a pair of specific primers and a corresponding probe were used to amplify JXwn06 nsp9 gene, and a standard curve was established for the nsp9 gene by plotting log_10_ copy number against the cycling threshold (C*_T_*) value (*γ*_JX_CT = −3.234x + 40.043).

### Statistical analysis

GraphPad Prism version 7.0 (La Jolla, CA, United States) was used to perform the statistical analyses (two-tailed unpaired Student’s *t*-tests). Asterisks indicate statistical significance; ns, no significance; *p < 0.05, **0.001 < *p* < 0.01, ***p < 0.001, ****p < 0.0001. Error bars indicate ± standard deviation (SD).

## Results

### Construction and recovery of PRRSV chimeric mutants

**Figure 1:**
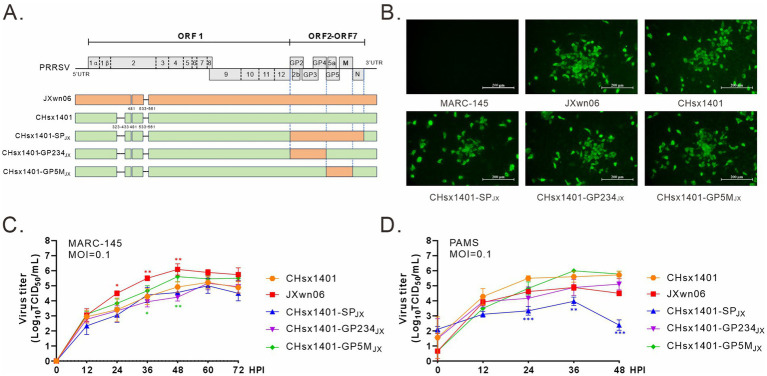
Construction and recovery of PRRSV chimeric mutants. **(A)** Genome organization and the constructs of PRRSV mutants; UTR, untranslated region. **(B)** Visualization of cytopathic effects (CPE) using IFA. MARC-145 cells in six-well plates were infected with the indicated viruses at an MOI of 0.1. At 24 h post infection (HPI), the cells were fixed and stained with PRRSV N protein antibody. CPE were visualized using a microscope. **(C,D)** Multiple-step growth analysis. MARC-145 cells **(C)** and PAMs **(D)** were infected with the indicated viruses at an MOI of 0.1. At the specified times intervals, the cells were harvested and subjected to titration by endpoint dilution assay in MARC-145 cells. Statistical analysis was performed using a two-tailed Student’s *t*-test, and error bars indicate means ± standard deviation (SD). Asterisks (*) indicate the statistical significance: *p < 0.05, **0.001 < *p* < 0.01, ***p < 0.001.

Our previous study has demonstrated that the region coding for PRRSV structural proteins (SP) can provide effective homologous immune protection against the challenge by HP-PRRSV JXwn06 strain. To further delineate the specific region involved inducing protective immunity, the NADC30-like strain CHsx1401 was used as a receptor to construct chimeric viral mutants using reverse genetics. The region encoding GP2/3/4 or GP5/M was replaced by that from JXwn06 to make respective chimeric mutants CHsx1401-GP234_JX_ and CHsx1401-GP5M_JX_ (Figure 1A). Additionally, a derivative (CHsx1401-SP_JX_) carrying only the structural protein (SP) region of the strain JXwn06 was used as a control (Kong et al., 2023a,b). All mutants were successfully recovered upon transfection of the infectious cDNA clone plasmids into MARC-145 cells, an *in vitro* supporting cell line, and the virus-induced CPE was monitored on a daily basis and characterized by cell detachment and clustering. These viruses were further confirmed via whole genome sequencing and IFA using anti-PRRSV N protein antibody in MARC-145 cells (Figure 1B). Next, the virus growth curve analysis was performed at an MOI of 0.1 in both MARC-145 cells and in porcine primary alveolar macrophages (PAMs). The results showed that the parental viruses JXwn06 and CHsx1401 grew differently in both cell types by that JXwn06 grew better than CHsx1401 in MARC-145 cells while the situation was reversed in PAMs. Exchange of the whole SP region (CHsx1401-SP_JX_) slightly decreased the replication of CHsx1401 in MARC-145 cells but significantly in PAMs. It seems the region for GP2/3/4 is the determinant as its substitution (CHsx1401-GP234_JX_), but not GP5/M (CHsx1401-GP5M_JX_), reduced the virus growth (Figures 1C,D).

### Pathogenic properties of PRRSV chimeric mutants

**Figure 2:**
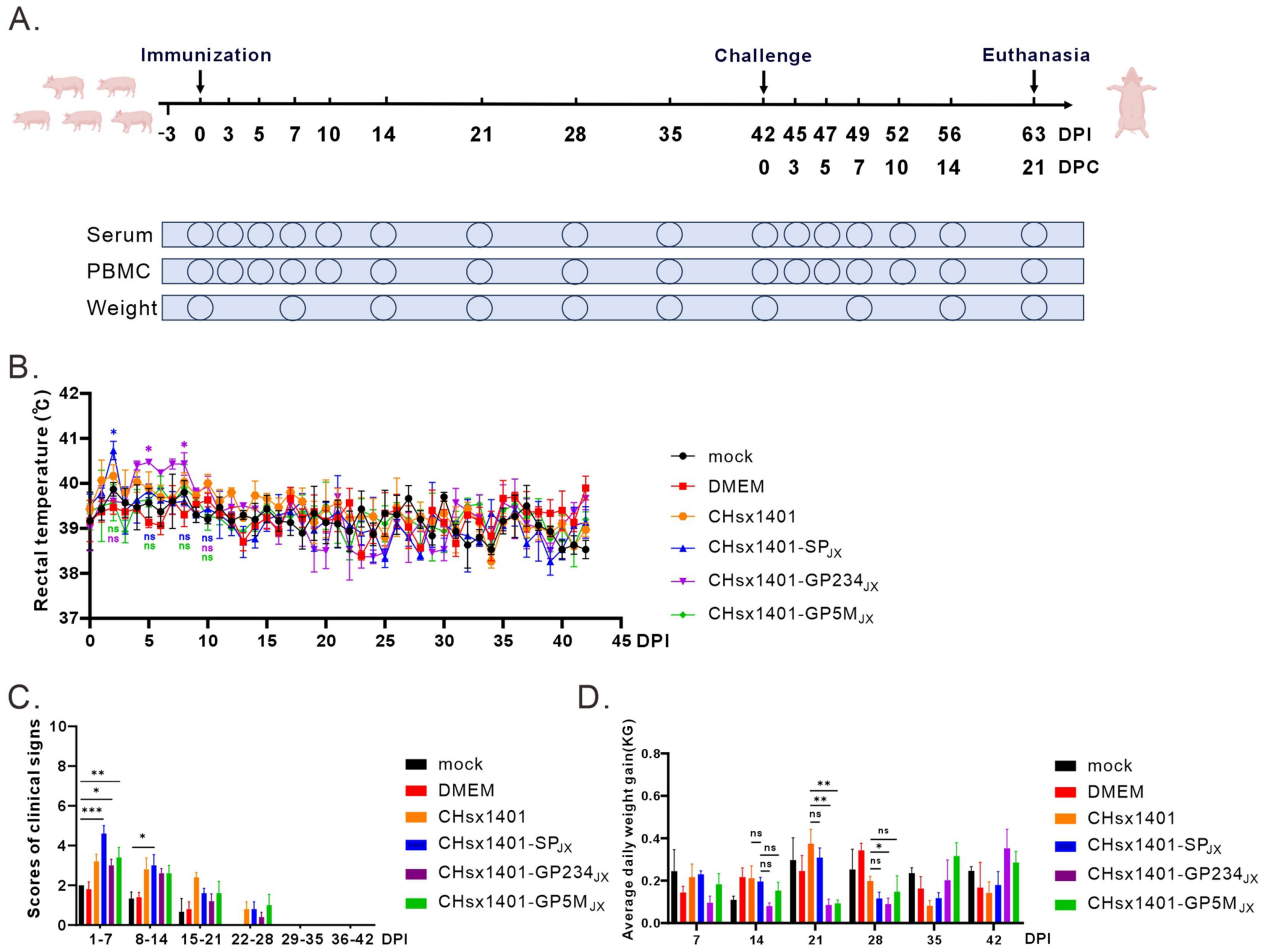
Pathogenicity analysis of PRRSV chimeras in piglets. **(A)** Scheme of the animal experiment protocol. **(B)** The rectal temperature curves of piglets after immunization with PRRSV chimeric mutants. **(C)** The clinical mental state scores of piglets. **(D)** Average daily weight gain of piglets. Total scores for each piglet represent the sum of the GCS, RCS, and NSS **(C,D)**. Statistical analysis was performed using a two-tailed Student’s t test, and error bars indicate means ± standard deviation (SD). Asterisks (*) indicate the statistical significance: *p < 0.05, **0.001 < *p* < 0.01, ***p < 0.001, ns, no significance.

To evaluate the pathogenic properties of these chimeric viruses, 28-day-old specific pathogen-free (SPF) piglets were randomly divided into 6 groups and intranasally immunized with indicated viruses at a dose of 2 × 10^5^ TCID_50_ or with DMEM medium as a negative control, and no treatment for mock group. The rectal temperature and clinical symptoms of piglets were monitored daily (Figure 2A). Consistent with previous report, piglets infected with the parental virus CHsx1401 exhibited a transient fever exceeding 40°C on 3 DPI (days post infection) and maintained a good appetite, whereas the group CHsx1401-SP_JX_ displayed elevated temperatures beginning from 1 DPI, peaking at an average of 40.6°C on 2 DPI, accompanied by depression and reduced appetite; however, their body temperature returned back to normal on day 4 DPI. Interestingly, the CHsx1401-GP234_JX_ group showed increased temperature (40.4°C) from 5 DPI and last for about 1 week, while the CHsx1401-GP5M_JX_ group developed slight fever on 6 DPI and, with a maximum average body temperature of approximately 40°C. Throughout the experimental process, individual piglets exhibited a loss of appetite during episodes of fever, which subsequently returned to normal levels (Figure 2B). Additionally, after chimeric viruses infection, all piglets exhibited a moderate coughing, sneezing, lethargic, and a decreased appetite. Among them, the CHsx1401-SP_JX_ group showed a strongest clinical symptom than other groups; and subsequently, appetite returned to normal and respiratory symptoms gradually abated after the temperature dropped at 28 DPI (Figure 2C). In comparison, CHsx1401-GP234_JX_ and CHsx1401-GP5M_JX_ groups displayed a slightly lower average daily gain (ADG) than CHsx1401 and CHsx1401-SP_JX_ groups during the 28 DPI period, whereas a higher ADG in CHsx1401-GP234_JX_ and CHsx1401-GP5M_JX_ groups than CHsx1401 and CHsx1401-SP_JX_ groups was assay after the 28 DPI period (Figure 2D).

### Viremia clearance analysis of chimeric mutants in piglets

**Figure 3:**
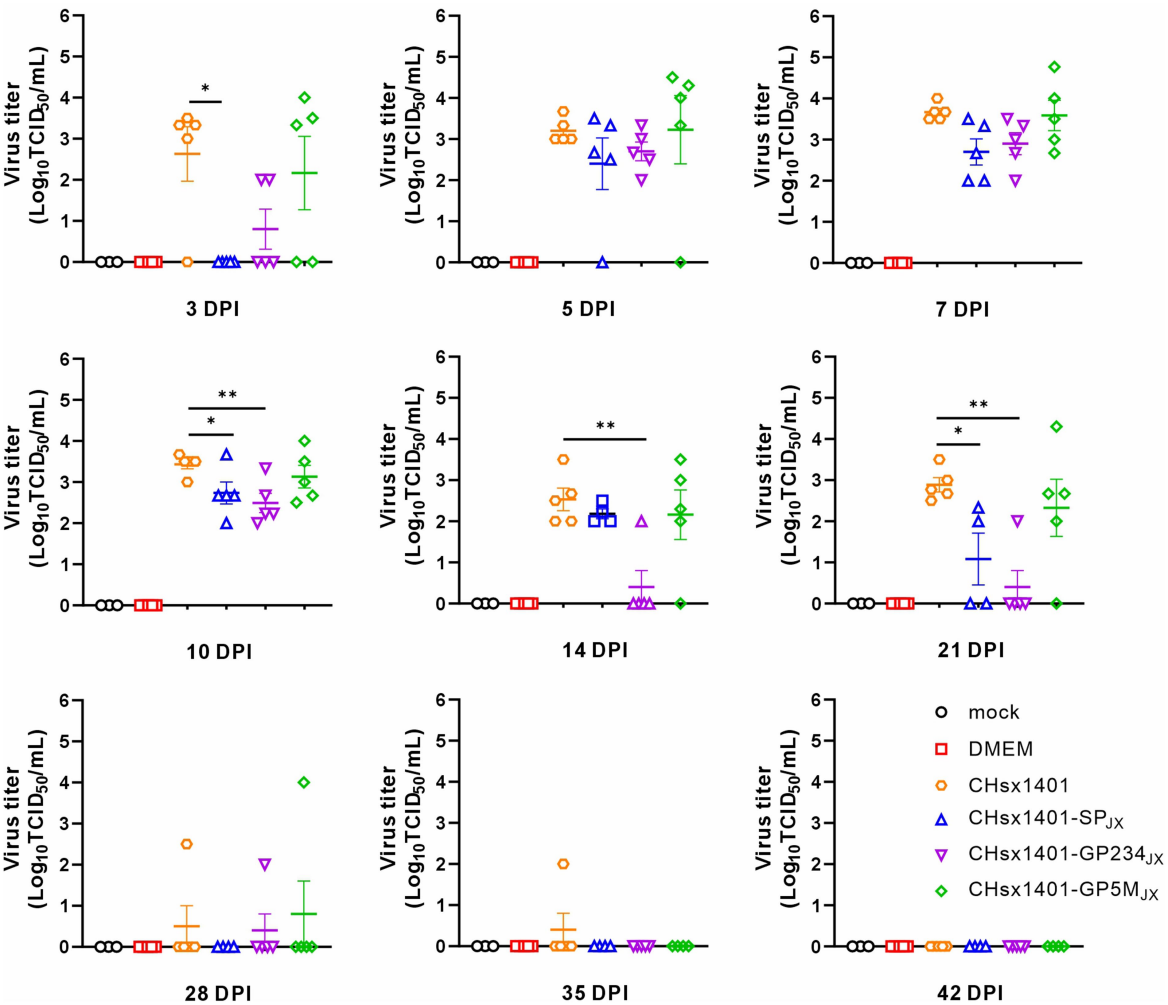
Viremia clearance analysis of chimeric mutants in piglets. The viral load in the blood harvested at various time intervals following infection was determined in MARC-145 cells using endpoint dilution assay. Statistical analysis was performed using a two-tailed Student’s *t*-test, and error bars indicate means ± standard deviation (SD). Asterisks (*) indicate the statistical significance: *p < 0.05, **0.001 < *p* < 0.01, ***p < 0.001.

The turnover of inoculated chimeric viruses in the blood was investigated. Except of the CHsx1401-SP_JX_ group, viremia was progressively detected in the other piglets at 3 DPI and became detectable for all piglets at 7 DPI. This trend persisted until the 14 DPI. Among these groups, the viral load in the blood of the CHsx1401 group displayed fastest rising, but was slowly and completely cleared until 42 DPI. The mutant CHsx1401-SP_JX_ exhibited the fastest declining kinetics as compared to other groups (Figure 3). The groups of CHsx1401-GP234_JX_ and CHsx1401-GP5M_JX_ showed intermediate phenotype; they reached peaks around 5 DPI-7 DPI, with viral clearance occurring first at 14 DPI for these groups, except for one piglet that cleared the virus at 28 DPI. Nevertheless, the overall clearing trend of CHsx1401-GP234_JX_ group was faster than that of the CHsx1401-GP5M_JX_ group. Both CHsx1401-GP234_JX_ and CHsx1401-GP5M_JX_ were completely cleared at 35 DPI.

### The structural proteins are crucial against the HP-PRRSV challenge

**Figure 4:**
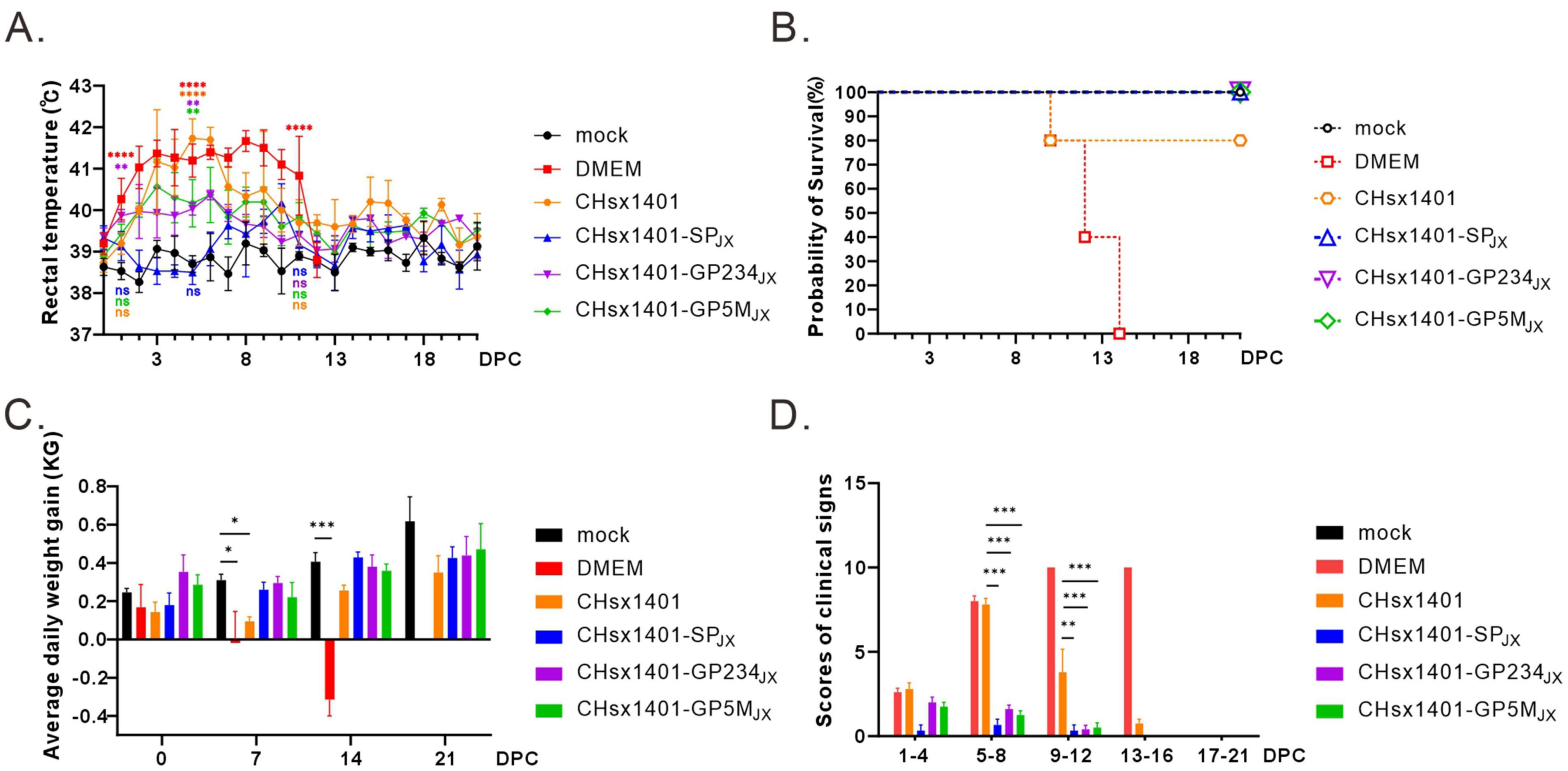
Structural proteins are crucial for the immune protection of HP-PRRSV JXwn06 challenge. **(A)** The rectal temperature fluctuates following JXwn06 challenge. **(B)** Survival curve. **(C)** The average daily weight gain of piglets after the JXwn06 challenge. **(D)** Clinical mental state scores of different groups. The clinical scoring included the gross clinical score (GCS), respiratory clinical score (RCS), and nervous signs score (NSS). Total scores for each piglet represent the sum of the GCS, RCS, and NSS. Statistical analysis was performed using a two-tailed Student’s *t*-test, and error bars indicate means ± standard deviation (SD). Asterisks (*) indicate the statistical significance: *p < 0.05, **0.001 < *p* < 0.01, ***p < 0.001, ****p < 0.0001; ns, no significance.

To evaluate the immunoprotective effect of chimeric viruses, all piglets were challenged with the wild-type JXwn06 at 42 DPI, a time point when viremia of all infected piglets became undetectable. As expected, the groups immunized with CHsx1401 and DMEM exhibited the quickest and most notable increase in body temperature, reaching a peak of approximately 41.7°C. This significant rise was accompanied by severe respiratory symptoms, as evidenced by significant signs of coughing, nasal discharge, labored breathing, and a lack of appetite. The DMEM group experienced cyanotic ears, depression, and lost appetite at 7 DPC, and all piglets eventually died at 14 DPC after 10 days of elevated high temperatures. However, the body temperatures of 80% piglets in CHsx1401 immunization group returned to normal and 20% piglets also experienced death (Figures 4A,B). On the other hand, all the piglets in the groups CHsx1401-GP234_JX_ and CHsx1401-GP5M_JX_ survived the challenge, but some pigs experienced a transient increase of the body temperatures to 40.5°C for several days before returning back to baseline. Interestingly, the group CHsx1401-GP5M_JX_ showed longer prolonger higher temperatures than the group CHsx1401-GP234_JX_ (Figure 4A). In contrast, the group CHsx1401-SP_JX_ showed normal temperature fluctuation during the course of challenge and all pigs survived. Overall, there was no significant difference in ADG among CHsx1401-GP234_JX_, and CHsx1401-GP5M_JX_ immunization groups (Figure 4C), as evidenced by the piglets displaying good mental condition and normal appetites (Figure 4D).

**Figure 5:**
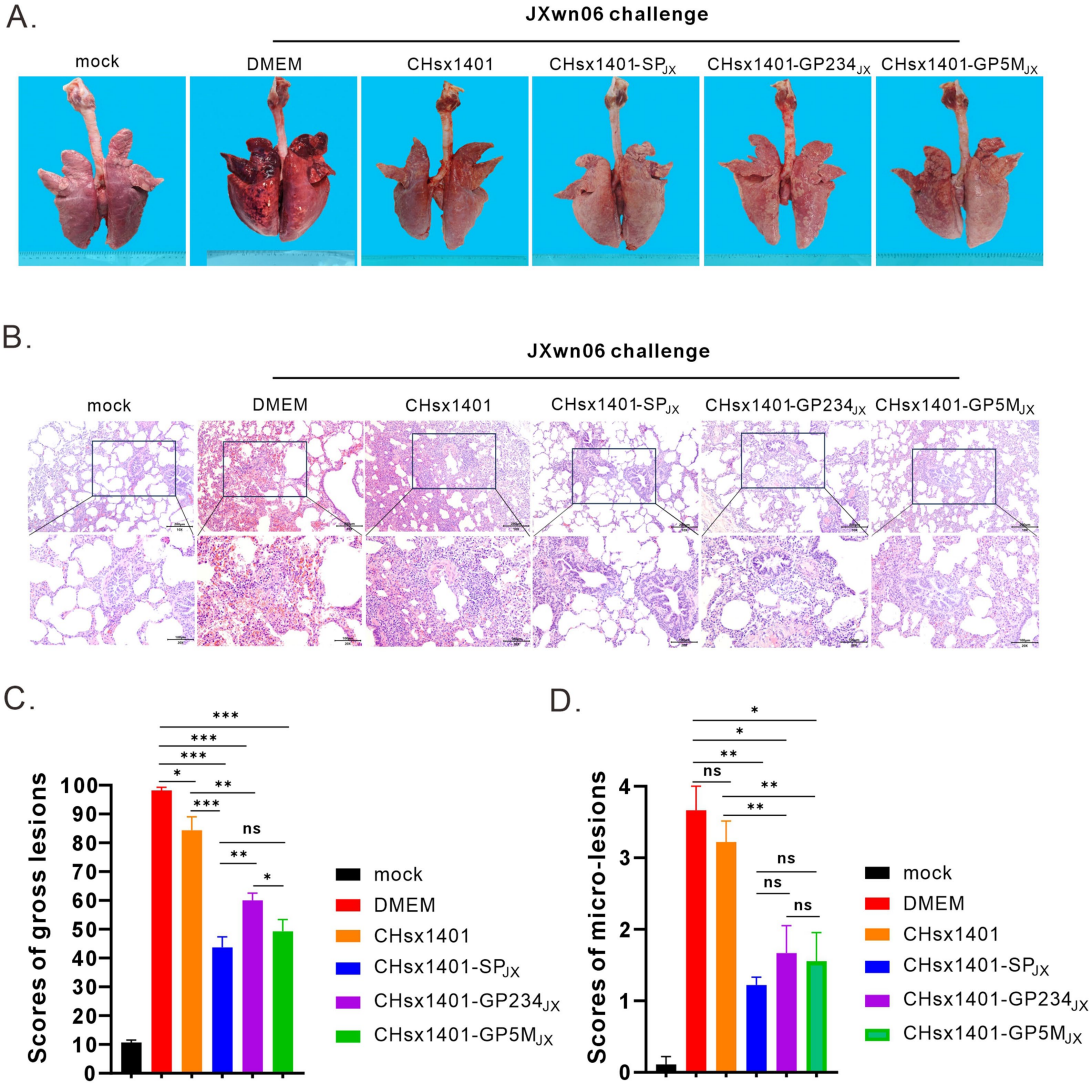
Evaluation of lung lesions and viral tissue load. **(A)** Representative gross lung lesions of each group at 21 DPC following JXwn06 challenge. **(B)** Representative images of microscopic lung lesions (H&E staining) at 21 DPC following JXwn06 challenge. **(C,D)** Mean scores of gross lesions **(C)** or microscopic lung lesions **(D)** of each group. Statistical analysis was performed using a two-tailed Student’s *t*-test, and error bars indicate means ± standard deviation (SD). Asterisks (*) indicate the statistical significance: *p < 0.05, **0.001 < *p* < 0.01, ***p < 0.001; ns, no significance.

All the piglets were euthanized and necropsied at 21 DPC. Postmortem examination revealed that, in comparison to the mock group, the DMEM and CHsx1401 group exhibited the most pronounced lung lesions were, displaying varying degrees of pulmonary damage; the CHsx1401-SP_JX_ group displayed the normal or very minor lesion lesions, whereas the CHsx1401-GP5M_JX_ and CHsx1401-GP234_JX_ groups showed the intermediate phenotype (Figures 5A,C). A similar trend was also observed for the microscopic lesions by hematoxylin and eosin (H&E) staining of lungs (Figures 5B,D). The JXwn06 induced much more severe lung injury in DMEM and CHsx1401 immunization groups; with typical lesions that are often seen in HP-PRRSV-infected piglets, such as alveolar hemorrhage, inflammatory cell infiltration, and pulmonary congestion. However, these microscopic symptoms are rarely observed in CHsx1401-SP_JX_, CHsx1401-GP234_JX_, and CHsx1401-GP5M_JX_ immunization groups (Figures 5B,D).

### Structural proteins contribute to the clearance of the strain JXwn06 in the piglets

**Figure 6:**
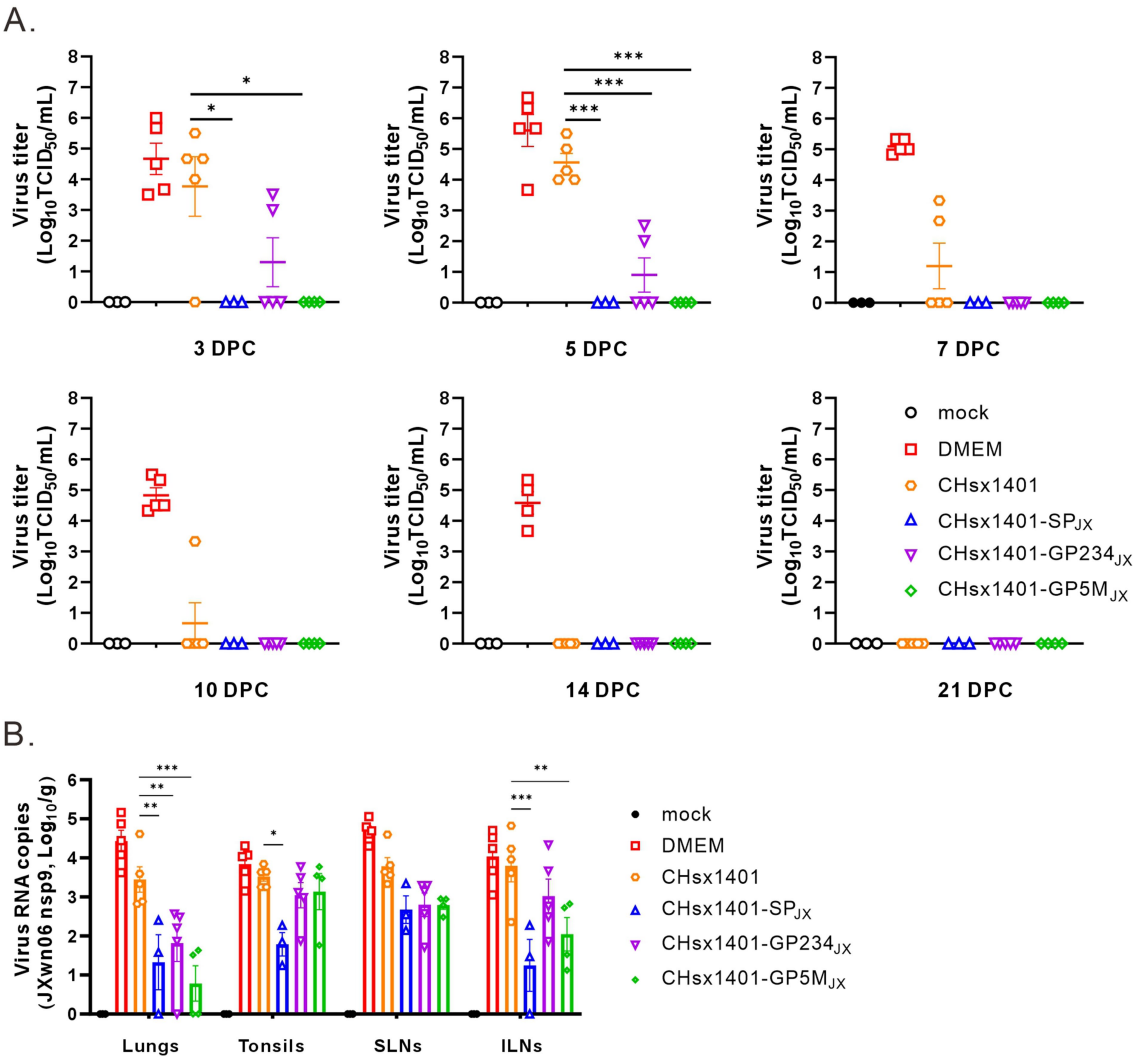
The structural proteins contribute to the clearance of the JXwn06 strain in the blood and the tissues of piglets. **(A)** Viremia status in the piglets following JXwn06 challenge. **(B)** Viral loads of JXwn06 strain in lungs and secondary lymphoid tissues were determined using RT-qPCR. Statistical analysis was performed using a two-tailed Student’s *t*-test, and error bars indicate means ± standard deviation (SD). Asterisks (*) indicate the statistical significance: *p < 0.05, **0.001 < *p* < 0.01, ***p < 0.001.

Next, the effect on viral persistence in both blood and tissues was also assessed. The piglets in the groups DMEM and CHsx1401 mounted a significant viremia following challenge by JXwn06 that peaked at around 3 DPC-5 DPC, and the viremia for the group CHsx1401 were not cleared until 14 DPC. In contrast, all three chimera groups showed low level of viremia that were quickly cleared from the blood at 5 DPC-7 DPC. Specifically, the viremia was not detectable at 3 DPC for the groups CHsx1401-SP_JX_ and CHsx1401-GP5M_JX_, suggesting that the region for GP5/M plays a significant role for viremia clearance (Figure 6A).

We next collected the tissues, including lungs, tonsils, inguinal lymph nodes (ILNs), and submandibular lymph nodes (SLNs) and processed individually for quantitative analyses of viral tissue load by absolute real-time reverse transcription PCR (RT-qPCR) by targeting the nsp9-coding region to differentiate CHsx1401 or its derivatives from JXwn06. Except for the mock control, viral RNAs could be detected in all tissues, but all four immunization groups had the ability to reduce to the viral tissue load of JXwn06 in tissues to varying extents (Figure 6B). Overall, the three chimeric groups showed a better reduction of viral tissue load than the CHsx1401 group, whereas the CHsx1401-SP_JX_ and CHsx1401-GP5M_JX_ groups exhibited the most pronounced reduction of viral load, achieving a decrease by approximate 100 ~ 1,000 fold in the lung and 100 fold in the ILNs compare to DMEM group. As analyzed for the three chimeric groups, CHsx1401-SP_JX_ showed a slightly better or comparable effect for viral tissue load reduction than the groups CHsx1401-GP234_JX_, and CHsx1401-GP5M_JX_ (Figure 6B). Together, the above findings suggest that the region for GP5 and M proteins has a greater efficiency in clearing the virus form the blood and tissues when compared to the region for GP2/3/4, but the best efficiency requires the whole region of SP.

### The effect of chimeric viruses on humoral immunity

**Figure 7:**
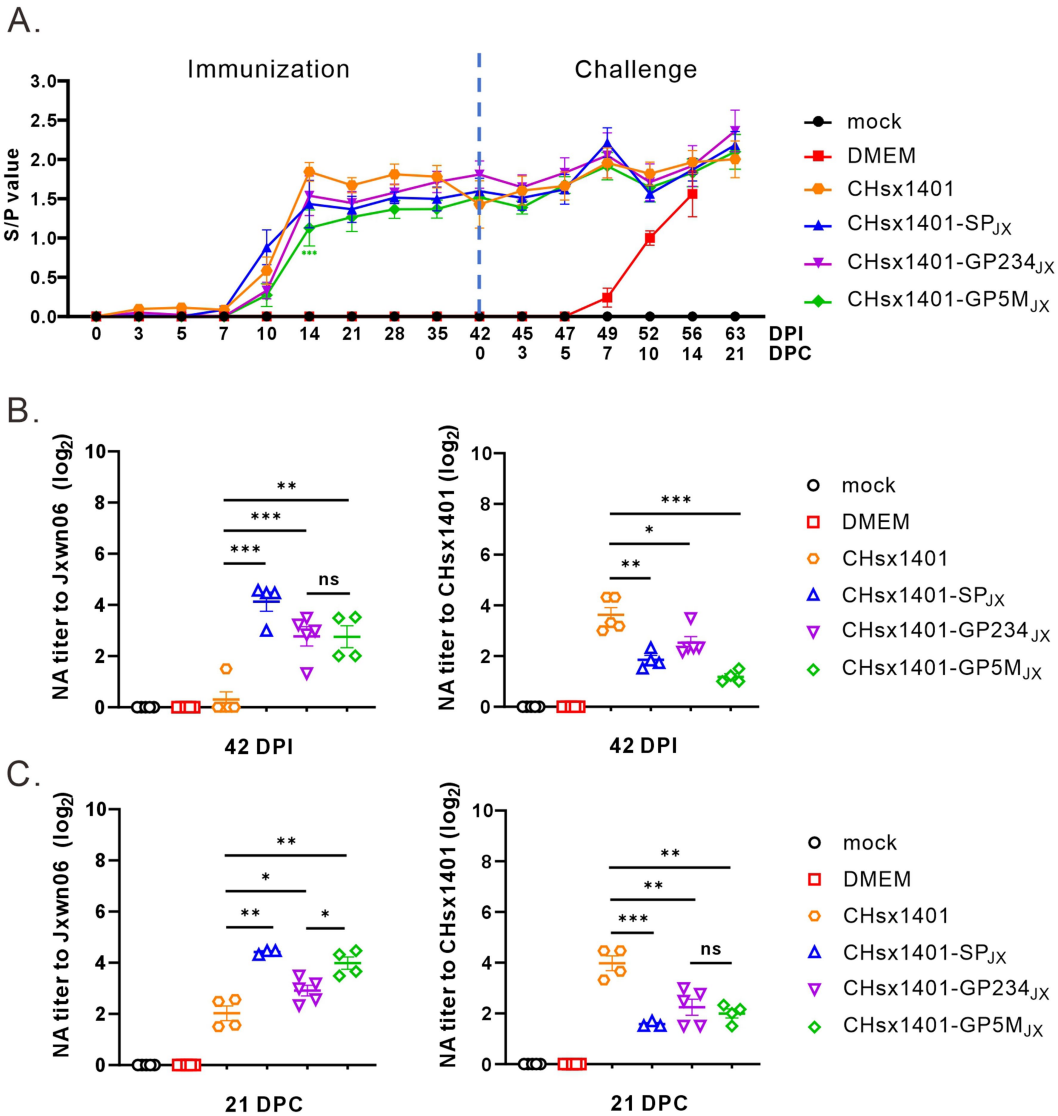
The effect of exchange of SP on humoral immunity. **(A)** The levels of PRRSV N protein antibodies. **(B,C)** The neutralizing antibodies (NA) titer in the serum at 42 DPI **(B)** or 21 DPC **(C)** after immunization. Error bars represent standard deviation (SD). Asterisks (*) mean the statistical difference: ns, no significance; *p < 0.05, **0.001 < *p* < 0.01, ***p < 0.001, ns, no significance.

To examine the humoral immune response induced by chimeric viruses, the serum samples from piglets were collected at indicated time points for analyzing the level of PRRSV antibodies by JNT Porcine reproductive and respiratory syndrome virus (PRRSV) AB ELISA kit. The PRRSV antibodies of all piglets became positive at 10 DPI. Although no significant differences were noted among the various groups, CHsx1401-GP5M_JX_ induced a slightly lower humoral immune response than that of parental viruses CHsx1401. Notably, a discernible trend of antibody re-elevation was evident in all groups after wild-type JXwn06 challenge (Figure 7A).

Subsequently, the neutralization abilities (NA) of antibodies against the CHsx1401 and JXwn06 viruses were evaluated at 42 DPI and 21 DPC in MARC-145 cells. At 42 DPI (before JXwn06 challenge), the neutralizing antibodies generated by CHsx1401 exhibit a pronounced neutralizing ability against the CHsx1401 strain, but almost no neutralizing ability against the JXwn06 strain; however, the neutralizing antibodies induced by CHsx1401-SP_JX_, CHsx1401-GP5M_JX_ and CHsx1401-GP234_JX_ showed substantial neutralizing efficacy against both CHsx1401 and JXwn06 strains. There was no statistically significant difference observed among CHsx1401-SP_JX_, CHsx1401-GP5M_JX_ and CHsx1401-GP234_JX_ in terms of their neutralizing antibody responses against either strain (*p* > 0.05) (Figure 7B). At the 21 DPC, the levels of neutralizing antibody against CHsx1401 elicited by the chimeric virus did not exhibit a significant increase compared to the levels observed at 42 DPI (before JXwn06 challenge); however, there was a slight increase in the neutralizing antibody titer against JXwn06 relative to the levels at 42 DPI. Among the three chimeric viruses, CHsx1401-SP_JX_ elicited the highest level of neutralizing antibodies against JXwn06, followed by CHsx1401-GP5M_JX_, and CHsx1401-GP234_JX_ was the weakest (*p* < 0.05) (Figure 7C).

## Discussion

Rapid evolution of PRRSV in the field represents a major challenge for PRRSV-associated disease control and prevention. The high genetic variability has rendered current MLV vaccines less efficient to provide cross-protection against the challenge of various field strains (Zhang et al., 2023). Hence, looking for the viral genetic determinants for inducing homologous immunity has been the focus for the PRRSV community. Our recent studies by making chimeric mutants have demonstrated that the viral structural protein-coding region (SP) is responsible for inducing homologous protective immunity. In this study, we went further to show that the regions for GP5/M and for GP2/3/4 play a synergistic role in inducing protective immunity and the best efficacy requires the whole SP region. The relevant significance and insights are discussed below.

The past 30 years have seen extensive efforts to understand the PRRSV-induced homologous and heterologous immunity or develop vaccines with broad cross-protection efficacy by making chimeric PRRSV mutants via exchange of certain specific SP regions or synthesized consensus sequences of different PRRSV strains (Tian et al., 2017; Park et al., 2021). So far, diverse viruses have been used as the acceptor (backbone), and they are from several PRRSV-2 lineages (L1, L5, and L8), and these include VR-2332 (Sun et al., 2016), FL12 (Vu et al., 2015; Shabir et al., 2016; Jeong et al., 2021; Choi et al., 2021), JSTZ1712-12 (HP-PRRSV, L8) (Chen et al., 2021; Li et al., 2022), GD (HP-PRRSV) (Li et al., 2024), Ingelvac PRRS MLV (Ellingson et al., 2010), VR2385 (Tian et al., 2017; Ni et al., 2013), and APRRS (very close to Primevac vaccine) (Li et al., 2008), and some from PRRSV-1 [SD01-08 (European PRRSV)] (Kimpston-Burkgren et al., 2017). Despite the result variations from different experiments from different laboratories, the results generate lots of useful information for understanding viral pathogenesis and cross-protection immunity. Overall, it is clear that the SP region plays an important role in inducing homologous immunity, although a definitive role of specific SP region is still under some debate, and the genetic variations often lead to reduce or poor cross protection efficiency or poor viral clearance, the consequence of which frequently results in generation of viral recombinants, leading to PRRS outbreaks (Li et al., 2017; van Geelen et al., 2018). However, there are two unfortunate caveats in most of these studies: either the viruses used for the challenge experiment are not virulent enough to cause the pig death, or the acceptor and challenge viruses are not heterologous enough. In this situation, it is necessary to use a highly virulent virus as a challenge model organism to further investigate the role of viral structural protein in induction of protective immunity. Hence, we used two genetically distant virus, NADC30-like strain CHsx1401 (lineage 1) and HP-PRRSV JXwn06 (lineage 8), as the respective acceptor and donor viruses. Genetically, their structural protein coding regions show a difference of about 28.25% at amino acid level. Moreover, we recently showed that immunization of piglets with CHsx1401 does not provide efficient protection against the challenge by HP-PRRSV strain JXwn06.

Our assessment started with a previous study by construction of chimeric viruses via substitution of SP of CHsx1401 with that of JXwn06 in the backbone of CHsx1401 to generate mutant CHsx1401-SP_JX_ (Kong et al., 2023a). It has been showed that immunization of pigs with CHsx1401-SP_JX_ can protect pigs from lethal challenge against JXwn06. The objective of this study was to further assess the individual role of the minor structural proteins GP2/3/4 and the major structural proteins GP5/M in induction of protective immunity. The challenge model was quite successful as the pigs in the group CHsx1401-SP_JX_ all survived the lethal challenge by JXwn06 and exhibited normal temperate fluctuation whereas the death rate in the DMEM group was 100% and all the pigs died within 14 DPC. One significant finding from this experiment is that the exchange of either GP2/3/4 or GP5/M region alone is sufficient to provide clinical protection against the lethal challenge by JXwn06 in terms of the pig survival rate. In addition, immunization with the two chimeras significantly reduced clinical sign and tissue injury. It should be noted that some tissue damages in the lungs can still be seen in the chimera groups; however, these can be due to that the viruses used for immunization still retain virulence and could cause damage during the immunization stage. The second interesting finding is that GP5/M region appears to play a significant role in viremia clearance, and the pigs in this group did not mount an effective viremia. This result is also consistent with a previous observation (Sun et al., 2016). In contrast, two pigs in the GP2/3/4 group showed certain amount of viremia. Overall, the chimera groups exhibited a higher efficient and much faster viremia clearance than the CHsx1401 group. It should be noted that, despite great genetic difference, the immunization with CHsx1401 can provide partial protection as 80% of the pigs survived the lethal challenge and immunized pigs had a faster viremia clearance kinetics than the DMEM group, although much slower than the chimera groups.

In conclusion, the results in this report suggest that the SP region as a whole performs better than either GP2/3/4 or GP5/M region alone in induction of homologous immunity, whereas either of two regions can induce sufficient amount of protective immunity. These findings have important implications for understanding protective immunity and in further vaccine design and development.

## Data Availability

The original contributions presented in the study are included in the article/Supplementary material, further inquiries can be directed to the corresponding authors.
