# Supplementary material for: The role of major and minor structural proteins of porcine reproductive and respiratory syndrome virus in induction of protective immunity
Source: Front Microbiol. 2025 Mar 19;16:1563186. doi: 10.3389/fmicb.2025.1563186 (PMC11961951; doi:10.3389/fmicb.2025.1563186)
Supplement: Supplementary file 1 [file Data_Sheet_1.PDF]

Table S1. The primers used for construction of chimeric viruses

Primer Name	position	Sequences (5'-3')
Up-GP234sx-1F	11566-11603[b]	CAATGAAGCGTTTCGTGCGCGCC(*Asc* I)AGAAAGGGAAGATTT
Up-GP234sx-1R	11666-11681[b]	TAGACCCCATTTCATTTCAGTTCAAGCCTAA
GP234$_{JX}$-2F	11984-11998[a]	TTAGGCTTGAACTGAAATGAAATGGGGTCTA
GP234$_{JX}$-2R	13674-13688[a]	GCATTTCCCCAACATATCTAAACATTCAAATTGCCAGTAG
Down-GP234sx-3F	13387-13411[b]	ATGTTTAGATATGTTGGGGAAATGCCTACTGGCAATTTGA
Down-GP234sx-3R	15045-15082[b]	GGGACCATGCCGGCCTTAATTAA(*Pac* I)TTTTTTTTTTTTTTT
Up-GP5Msx-1F	11566-11603[b]	CAATGAAGCGTTTCGTGCGCGCC(*Asc* I)AGAAAGGGAAGATTT
Up-GP5Msx-1R	13372-13396[b]	GCACTTCCCCAACATATCTAAACATTCAAATTGCCAACAG
GP5M$_{JX}$-2F	13699-13713[a]	GGCAATTTGAATGTTTAGATATGTTGGGGAAGTGC
GP5M$_{JX}$-2R	14781-14810[a]	TGCTGTCTGCCGTTGTTATTTGGCATATTTAACAAGGTTTACCAC
Down-GP5Msx-3F	14498-14523[b]	CCTTGTTAAATATGCCAAATAACAACGGCAGACAGCA
Down-GP5Msx-3R	15045-15082[b]	GGGACCATGCCGGCCTTAATTAA(*Pac* I)TTTTTTTTTTTTTTT

F: forward primer; R: reverse primer

[a]Genome position is based on Gene Bank submission EF641008.1 (JXwn06).

[b]Genome position is based on Gene Bank submission KP861625.1 (CHsx1401).

Underlining indicates enzyme restriction sites.

“Up” or “Down” is the upstream or downstream sequence fragment representing the target gene.
