# Supplementary material for: The role of major and minor structural proteins of porcine reproductive and respiratory syndrome virus in induction of protective immunity
Source: Front Microbiol. 2025 Mar 19;16:1563186. doi: 10.3389/fmicb.2025.1563186 (PMC11961951; doi:10.3389/fmicb.2025.1563186)
Supplement: Supplementary file 2 [file Data_Sheet_2.PDF]

Table S2. The primer sequences of JXwn06 nsp9 gene used for qPCR

Primer Name	Sequences (5'-3')
nsp9-JXwn06-F	CTGCGATCGATCCACACCTG
nsp9-JXwn06-R	GCGTGACCAGTAAGTCGTGG
nsp9-JXwn06-probe	VIC-TTTGCCGCCAATCTTCTT-MGB

F: forward primer; R: reverse primer
